# Harnessing Artificial Intelligence to Code a Decision-Making Aid for the Prostate Cancer Brachytherapy Multidisciplinary Meeting Using Retrieval-Augmented Generation

**DOI:** 10.7759/cureus.103457

**Published:** 2026-02-12

**Authors:** Abraham Gabriel, Georgios Antonoglou, Stephen Langley, Joseph Gabriel

**Affiliations:** 1 Medicine, Ashford and St. Peter’s Hospitals NHS Foundation Trust, Surrey, GBR; 2 Urology, Royal Surrey County Hospital, Guildford, GBR; 3 Urology, Imperial College Healthcare NHS Trust, London, GBR; 4 Urology, Chelsea and Westminster NHS Foundation Trust, London, GBR

**Keywords:** artificial intelligence, brachytherapy, chatgpt, large language model, multidisciplinary team meeting, prostate cancer

## Abstract

Introduction

We have previously published the first application of artificial intelligence (AI) to streamline the real-world multidisciplinary meeting (MDM) process, finding that ChatGPT (OpenAI) could accurately recommend a treatment option in perfect concordance with the European Association of Urology (EAU) guidelines. ChatGPT now allows users to formally create their own generative pretrained transformer (GPT) tailored to a specific role using retrieval-augmented generation (RAG).

In this paper, we seek to utilize this technology for the first time in the prostate cancer multidisciplinary team (MDT) setting, to create a custom decision-making aid GPT for prostate cancer patients being treated with brachytherapy.

Methods

ChatGPT 4.0 was prompted to create a decision-making custom GPT using RAG to select brachytherapy treatment options for prostate cancer patients. While international guidelines exist, we opted to use our well-established local Royal Surrey guidelines as prior knowledge to the custom GPT.

Results

The custom GPT created is available on GitHub. Forty patients were discussed, with the AI-generated tool suggesting the same brachytherapy treatment strategy as that of the real-world MDM in all (40/40) cases.

Conclusion

We believe that this simple study has demonstrated a very practical application of this novel AI technology. This has the capacity to reinvent the paradigm in the preparation of patient cases prior to a cancer board or MDM, as cases can be preselected for the optimal treatment strategy with 100% accuracy prior to discussion by clinicians in the MDM, streamlining the process and facilitating a nuanced discussion by eliminating irrelevant treatment strategies beforehand.

## Introduction

The use of artificial intelligence (AI) in the form of large language models (LLMs) in medicine has surged in the last two years, with interest largely fueled by the meteoric rise of ChatGPT (OpenAI), released on November 30, 2022 [1]. In a recent survey by Wiley, 81% of academics said they had made use of it personally or professionally [2]. Recent applications in urology have focused on its use in patient education and assisting urologists in accessing and summarizing clinical guidelines, with an increasing awareness of the ethical and legal implications [3-7].

We have previously published the first potential application of ChatGPT 4.0 in the prostate cancer multidisciplinary meeting (MDM) setting, exploring a novel application of AI to streamline the real-world MDM process, finding that ChatGPT could accurately recommend a treatment option in perfect concordance with the European Association of Urology (EAU) guidelines [8]. ChatGPT now allows users to formally create their own generative pretrained transformer (GPT) tailored to a specific role using retrieval-augmented generation (RAG), in which specialized knowledge is first given to the model from authoritative sources.

One area of potential interest is the prostate cancer MDM meeting: in UK practice, the standard of care is for prostate cancer patients to be discussed in a multidisciplinary setting, where surgeons, oncologists, radiologists, pathologists, clinical nurse specialists and others collaborate together to ensure that best clinical practice is being followed by the individual clinician in charge of the patient’s care [9]. Such practice has been shown to improve clinical outcomes in prostate cancer [10]. MDMs are, however, resource-intensive, being both financially and time-costly, and improvements aimed at streamlining such meetings to run as efficiently as possible can make a significant improvement in resource allocation [11]. AI strategies may be the key to this.

In this paper, we seek to utilize this technology for the first time in the prostate cancer multidisciplinary team (MDT) setting, to further investigate if ChatGPT can create a custom decision-making aid GPT for prostate cancer patients being treated with brachytherapy, that may be used by MDT clinicians to recommend treatments.

## Materials and methods

ChatGPT 4.0 (OpenAI) was prompted on January 20, 2026 to create a decision-making aid custom GPT using RAG to select brachytherapy treatment options for prostate cancer patients. While international guidelines exist, we opted to use our well-established local Royal Surrey guidelines as prior knowledge to the custom GPT, used to treat the largest published cohort in the literature, with >5500 men over the past 25 years [12-15]. Forty consecutive patients listed for MDM discussion were selected. Code interpreter capabilities were enabled, allowing ChatGPT to write code and analyze uploaded files, and live web browsing was disabled to create a closed system.

The instructions were given to the GPT as follows, and an example is available to view in Appendix A.

Welcome to your specialized role as a Urology Expert GPT. The GPT is designed as an assistant for a urologist, specializing in selecting the optimal treatment option for prostate cancer patients undergoing brachytherapy.

**Step 1: Read and Interpret

Inputs will be in the form of unedited patient reports or vignettes of patients with prostate cancer. You will be required to read and interpret the patient report. From the report you will need to extract the following parameters:

1) Gleason score (3+3. 3+4, 4+3, 4+4, 4+5. 5+4 or 5+5)

2) PSA

3) Prostate volume

4) Stage (T1c, T2a, T2b, T3a, T3b, T4)

5) Percentage of prostate biopsy core positive

**Step 2: You Will Then Apply the Following Rules

1) HDR-brachytherapy (given in combination with EBRT and androgen deprivation treatment for 2-3 years) is only suitable for T3b disease.

2) LDR monotherapy is suitable for Gleason 3+3 disease or Gleason 3+4 disease if <50% of cores are positive, and PSA <15ng/ml and stage T1c, T2a or T2c. 3)

3) Large prostate >60cc on transrectal USS measurement require 3 months of androgen deprivation therapy before LDR brachytherapy (to reduce prostate volume). If Gleason 3+3 or Gleason 3+4 with <50% cores positive and a prostate >60cc, given LDR brachytherapy with 3 months neoadjuvant androgen deprivation therapy.

4) LDR brachytherapy in addition to androgen deprivation therapy for 3 months (before and after the LDR brachytherapy, 6 months total) is suitable for: Gleason 3+3 if ≥50% of cores involved, Gleason 3+4 if <50% of cores involved or if Gleason 4+3≥50% of cores involved, and PSA <15ng/ml and stage T1c, T2a, T2b or T2c.

5) LDR brachytherapy with EBRT and androgen deprivation treatment for 2-3 years combined is suitable for Gleason 4+3 with ≥50% cores involved, or any 4+4, 4+5, 5+4, and 5+5 disease, or PSA >15ng/ml, or T3a disease.

6) Brachytherapy is not suitable for T4 tumors, or in other scenarios that do not follow the above rules.

7) T3a disease does not require HDR-brachytherapy.

**Step 3 - Outputs: The Only Outputs You Can Produce as a Treatment Recommendation Are:

1) LDR monotherapy

2) LDR brachytherapy with 3 months neoadjuvant androgen deprivation therapy

3) LDR brachytherapy with external beam radiotherapy and androgen deprivation therapy for 6 months total.

4) LDR brachytherapy with external beam radiotherapy and androgen deprivation therapy for 2-3 years total.

5) HDR brachytherapy with external beam radiotherapy and androgen deprivation therapy or 2-3 years total.

6) Brachytherapy not suitable.

Inputted characteristics included Gleason grade (the standard grading system used in prostate cancer biopsy histology), tumor stage (as per TNM classification), prostate-specific antigen (PSA), prostate volume, and percentage of prostate biopsy cores positive for cancer [16-17]. In our center, brachytherapy is offered only in localized prostate cancer (N0) disease. The GPT was then tested by applying a variety of inputs to ensure functionality and was subsequently used during a real-world prostate brachytherapy MDM, in which patients’ parameters were entered into the tool simultaneously for discussion by clinicians. The GPT was not responsible for making the actual clinical decisions for patients, but its recommended treatments were compared to the actual MDT recommendations for concordance. All outputs from the GPT were manually evaluated against the real-world MDM by the authorship team.

## Results

The custom GPT .html calculator created can be located and downloaded at https://github.com/joegabrieldoctors/ProstateBrachytherapyGPT, with the interface visible in Appendix B. Forty consecutive patients were discussed, with the AI-generated tool suggesting the same brachytherapy treatment strategy as that of the real-world MDM in all (40/40) cases. Patient vignettes with all patient-identifiable information removed given to the model for assessment and its responses are tabulated in Table 1.

**Table 1:** Patient vignette presented to the custom generative pretrained transformer (GPT) created, its treatment recommendation, and those of the real-world multidisciplinary meeting (MDM). HDR: high-dose rate; LDR: low-dose rate; PSA: prostate-specific antigen; PV: prostate volume; G3+3: Gleason 3+3; G3+4: Gleason 3+4; G4+3: Gleason 4+3; G4+4: Gleason 4+4; T1c: stage T1c; T2: stage T2; T2a: stage T2a; T2b: stage T2b; T3: stage T3; T3a: stage T3a; T3b: stage T3b

Patient Vignette					Custom GPT Responses	Real-World MDM Recommendations
PSA (ng/ml)	PV (cc)	Gleason	T Stage	Cores Positive		
17	56	4+3	T3a	Not included	LDR brachytherapy with external beam radiotherapy and androgen deprivation therapy for 2-3 years total	LDR brachytherapy with external beam radiotherapy and androgen deprivation therapy for 2-3 years total
Not included	35	3+4	T2	4/16	LDR monotherapy	LDR monotherapy
28	Not included	5+5	T3a	Not included	LDR brachytherapy with external beam radiotherapy and androgen deprivation therapy for 2-3 years	LDR brachytherapy with external beam radiotherapy and androgen deprivation therapy for 2-3 years
6.7	56	3+4	T2	5/23	LDR monotherapy	LDR monotherapy
3.9	38	3+4	T2	12/19	LDR brachytherapy with external beam radiotherapy and androgen deprivation therapy for 2-3 years	LDR brachytherapy with external beam radiotherapy and androgen deprivation therapy for 2-3 years
7.9	25	3+4	T2b	14/20	LDR brachytherapy with external beam radiotherapy and androgen deprivation therapy for 6 months total	LDR brachytherapy with external beam radiotherapy and androgen deprivation therapy for 6 months total
15.0	43	3+4	T2	1/15	LDR brachytherapy with external beam radiotherapy and androgen deprivation therapy for 2-3 years	LDR brachytherapy with external beam radiotherapy and androgen deprivation therapy for 2-3 years
Not included	120	3+3	T2	Not included	LDR monotherapy with 3 months neoadjuvant androgen deprivation therapy	LDR monotherapy with 3 months neoadjuvant androgen deprivation therapy
11.1	34	4+3	T2	7/9	LDR brachytherapy with external beam radiotherapy and androgen deprivation therapy for 6 months total	LDR brachytherapy with external beam radiotherapy and androgen deprivation therapy for 6 months total
0.4	40	4+5	T3b	9/11	HDR brachytherapy with external beam radiotherapy and androgen deprivation therapy for 2-3 years	HDR brachytherapy with external beam radiotherapy and androgen deprivation therapy for 2-3 years
8.1	19	4+3	T2c	6/24	LDR brachytherapy with external beam radiotherapy and androgen deprivation therapy for 6 months total	LDR brachytherapy with external beam radiotherapy and androgen deprivation therapy for 6 months total
9.6	29	3+4	T2a	11/20	LDR brachytherapy with external beam radiotherapy and androgen deprivation therapy for 6 months total	LDR brachytherapy with external beam radiotherapy and androgen deprivation therapy for 6 months total
12.0	63	3+4	T2b	8/8	LDR brachytherapy with external beam radiotherapy and androgen deprivation therapy for 6 months total, with neoadjuvant androgen deprivation therapy for volume reduction	LDR brachytherapy with external beam radiotherapy and androgen deprivation therapy for 6 months total, with neoadjuvant androgen deprivation therapy for volume reduction
4.0	80	3+3	T2a	2/8	LDR monotherapy with 3 months neoadjuvant androgen deprivation therapy	LDR monotherapy with 3 months neoadjuvant androgen deprivation therapy
6.2	34	4+4	T4	3/9	Brachytherapy not suitable	Brachytherapy not suitable
16.8	45	4+5	T3b	9/10	HDR brachytherapy with external beam radiotherapy and androgen deprivation therapy for 2-3 years	HDR brachytherapy with external beam radiotherapy and androgen deprivation therapy for 2-3 years
13.2	54	3+4	7/10	T3a	LDR brachytherapy with external beam radiotherapy and androgen deprivation therapy for 2-3 years	LDR brachytherapy with external beam radiotherapy and androgen deprivation therapy for 2-3 years
11.4	70	4+3	T2c	8/10	LDR brachytherapy with external beam radiotherapy and androgen deprivation therapy for 6 months total, with neoadjuvant androgen deprivation therapy for volume reduction	LDR brachytherapy with external beam radiotherapy and androgen deprivation therapy for 6 months total, with neoadjuvant androgen deprivation therapy for volume reduction
9.1	49	4+3	T2b	7/7	LDR brachytherapy with external beam radiotherapy and androgen deprivation therapy for 6 months total	LDR brachytherapy with external beam radiotherapy and androgen deprivation therapy for 6 months total
13.5	55	3+4	T2c	10/15	LDR brachytherapy with external beam radiotherapy and androgen deprivation therapy for 6 months total	LDR brachytherapy with external beam radiotherapy and androgen deprivation therapy for 6 months total
7.5	85	3+4	T2a	4/20	LDR monotherapy with 3 months neoadjuvant androgen deprivation therapy	LDR monotherapy with 3 months neoadjuvant androgen deprivation therapy
8.5	44	4+3	T2a	6/14	LDR brachytherapy with external beam radiotherapy and androgen deprivation therapy for 6 months total	LDR brachytherapy with external beam radiotherapy and androgen deprivation therapy for 6 months total
11.0	40	3+3	T2b	4/18	LDR monotherapy	LDR monotherapy
9.8	52	3+4	T2a	3/14	LDR monotherapy	LDR monotherapy
10.3	38	3+4	T2b	12/18	LDR brachytherapy with external beam radiotherapy and androgen deprivation therapy for 6 months total	LDR brachytherapy with external beam radiotherapy and androgen deprivation therapy for 6 months total
6.4	75	3+3	T1c	1/10	LDR monotherapy with 3 months neoadjuvant androgen deprivation therapy	LDR monotherapy with 3 months neoadjuvant androgen deprivation therapy
5.6	31	4+4	T3b	11/12	HDR brachytherapy with external beam radiotherapy and androgen deprivation therapy for 2-3 years	HDR brachytherapy with external beam radiotherapy and androgen deprivation therapy for 2-3 years
12.5	90	3+4	T2a	3/10	LDR monotherapy with 3 months neoadjuvant androgen deprivation therapy	LDR monotherapy with 3 months neoadjuvant androgen deprivation therapy
7.3	28	4+4	T3b	4/8	HDR brachytherapy with external beam radiotherapy and androgen deprivation therapy for 2-3 years	HDR brachytherapy with external beam radiotherapy and androgen deprivation therapy for 2-3 years
12.4	45	4+3	T2c	6/12	LDR brachytherapy with external beam radiotherapy and androgen deprivation therapy for 6 months total	LDR brachytherapy with external beam radiotherapy and androgen deprivation therapy for 6 months total
9.5	39	3+4	T2b	10/14	LDR brachytherapy with external beam radiotherapy and androgen deprivation therapy for 6 months total	LDR brachytherapy with external beam radiotherapy and androgen deprivation therapy for 6 months total
7.1	42	3+3	T1c	2/14	LDR monotherapy	LDR monotherapy
18.1	48	3+4	T2b	6/10	LDR brachytherapy with external beam radiotherapy and androgen deprivation therapy for 2-3 years	LDR brachytherapy with external beam radiotherapy and androgen deprivation therapy for 2-3 years
7.7	43	4+3	T2a	3/12	LDR brachytherapy with external beam radiotherapy and androgen deprivation therapy for 6 months total	LDR brachytherapy with external beam radiotherapy and androgen deprivation therapy for 6 months total
5.4	40	4+4	T3b	10/11	HDR brachytherapy with external beam radiotherapy and androgen deprivation therapy for 2-3 years	HDR brachytherapy with external beam radiotherapy and androgen deprivation therapy for 2-3 years
17.2	36	3+4	T2b	6/10	LDR brachytherapy with external beam radiotherapy and androgen deprivation therapy for 2-3 years	LDR brachytherapy with external beam radiotherapy and androgen deprivation therapy for 2-3 years
6.9	47	3+4	T2a	4/18	LDR monotherapy	LDR monotherapy
9.2	74	3+3	T2b	2/10	LDR monotherapy with 3 months neoadjuvant androgen deprivation therapy	LDR monotherapy with 3 months neoadjuvant androgen deprivation therapy
12.7	41	4+3	T2c	5/12	LDR brachytherapy with external beam radiotherapy and androgen deprivation therapy for 6 months total	LDR brachytherapy with external beam radiotherapy and androgen deprivation therapy for 6 months total
5.4	40	4+4	T3b	10/11	HDR brachytherapy with external beam radiotherapy and androgen deprivation therapy for 2-3 years	HDR brachytherapy with external beam radiotherapy and androgen deprivation therapy for 2-3 years

## Discussion

We believe that this simple study has demonstrated a very practical application of this novel AI technology. In its latest ChatGPT 4.0 iteration, the LLM is immediately able to write functional code for decision-making tools that perfectly adhere to user-defined rules, such as those in a treatment algorithm for prostate cancer brachytherapy. We have previously demonstrated that it can be given real patient vignettes, taken directly from a real-world cancer MDM, and accurately summarize the best treatment options for individual patient’s parameters based on the recommendations in the latest EAU guidelines [8]. The present study takes this one step further, and the ease with which any user, with no coding experience, can harness ChatGPT to create a functional decision-making tool is remarkable. However, RAG has been used in recent studies investigating usage in patient information provision, to our knowledge, ours is the first study in the published literature using RAG in an MDM setting [18-19].

This has huge implications for the contemporary practice of MDMs and provides an opportunity for streamlining and speeding up such processes. It is feasible for patient vignettes to be analyzed in the manner we have demonstrated prior to a urology MDM by a referring hospital or MDM pathway coordinator, and for the range of treatment options it has recommended based on the guideline desired to be presented to clinicians, facilitating and speeding the decision-making process during these time-constrained meetings, with evidence showing that majority of MDM members favor processes to streamline these meetings [20]. It is particularly helpful in the setting of a prostate cancer brachytherapy MDT, in which specific modalities of brachytherapy (in combination with other options such as external beam radiotherapy, androgen deprivation therapy, or bladder outflow surgery) are administered according to local practice. Such binary rules and treatment recommendations are ideal for an LLM, allowing it to consistently recommend set treatments without variation, and potentially reducing the scope for the GPT to hallucinate [21]. When using RAG, a custom GPT will consult the rules and parameters set, in this case, our local brachytherapy guidelines, prior to any new generative response; we opted to use the GPT to code a .html decision-making aid, to allow consistency, ease of use, and to minimize any hallucinations or confabulations from outputs from the GPT directly.

We are not suggesting that ChatGPT can in any way take the place of a clinician in recommending treatment options; such decisions are nuanced and must be patient-specific. It is, however, undeniable that such widely available technology can and now should be used as tools by urologists and clinicians to enhance and assist our systems. Models such as ours could save valuable time spent on administrative tasks to free clinicians for more high-priority tasks in patient care. As LLMs’ usage becomes more available and AI takes on a greater role in our healthcare systems, it is becoming increasingly clear that clinicians who wish to remain ahead must become more familiar with these tools in order to remain at the forefront. Our study has demonstrated the ease with which a physician without any coding experience can harness LLM’s capabilities in patient care, with ChatGPT being the first to democratize the technology.

Limitations of our study include the small numbers tested within this proof-of-concept study and our use of only local guidelines as our use-case. We opted to use our own published local guidelines, as these are pertinent to a local use-case specific to our own center; further research could expand a model programmed to follow multiple local and national/international guidelines if desired. Another limitation of GPT technologies is overfitting, in which the model rigidly applies rules at the expense of the “softer” clinical decision-making nuance of clinicians for individual patients; we fully support clinical decision-making to ultimately be made by the clinician, and tools such as ours used only as aids to increase efficiency and streamlining. Our GPT focused only on textual inputs to select the correct treatment combination based on patient and disease characteristics; further scope could be made for ultrasound or MRI images of the prostate to be processed to allow for more detailed decision-making in actual brachytherapy delivery. Automation bias, in which a GPT model is relied upon when the inputs given to it are outdated, must also be safeguarded against, with regular updates required as guidelines change. Further validation is welcome with our code open-access.

As others have pointed out, it is imperative that uniform guidelines are established to inform the ethical and optimal use of LLMs in the research and healthcare settings [22]. Serious unanswered questions remain on a societal and legal level as to the processing, storage, and sharing of individual personal healthcare data on AI servers belonging to large multinational corporations.

We must also ensure that legal concerns regarding privacy and data protection are considered and regulated. As key stakeholders in the care of our patients, urologists should be involved and consulted in these regulatory processes.

It is clear that the blistering advances in LLM’s capabilities are outstripping our research output exploring them, and consequently, on our utilization of them clinically on a daily basis. ChatGPT can analyze and interpret images and conduct literature searches, and can now even talk, recognizing speech and responding directly to users, with potential applications for direct live translation between clinicians and patients [23-24]. The future is here with AI, and we ignore it at our own peril: urologists and oncologists must become more au fait with these tools for the betterment of our healthcare systems and patient care.

## Conclusions

Our study has demonstrated a use-case for a custom large language model in streamlining cases before an MDM. The tool built has the capacity to, with complete accuracy, predict the real-world treatment strategy that a prostate cancer MDM made. The further democratization of AI tools, such as this RAG of a large language model, allows a clinician without a technical background in coding to make a meaningful tool that can assist in speeding up clinical decision-making. This has untold potential benefits of optimizing physician time and clinician decision-maker’s time, and reducing administrative burden.

The necessary regulatory, legal, and ethical frameworks need to be streamlined to allow patient data to be utilized safely and securely in custom AI models, and it is imperative that urologists maintain their position as key stakeholders in these discussions, alongside computer scientists and lawmakers.

## Figures and Tables

**Figure 1:**
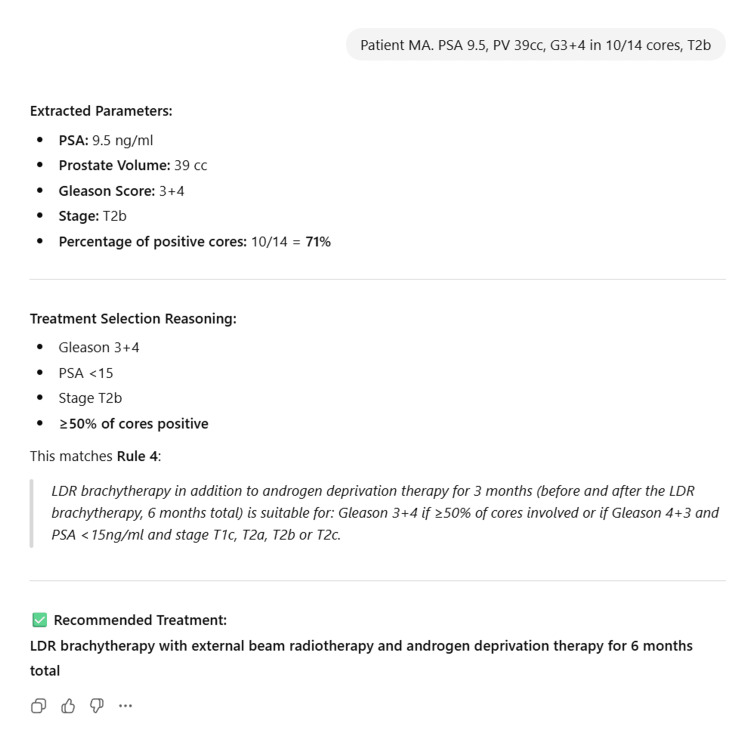
The interface of the GPT being given a patient vignette and its processing and output.

**Figure 2:**
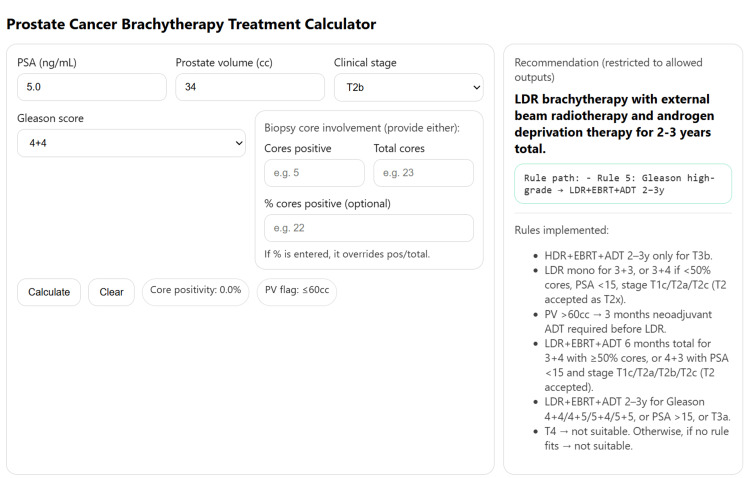
Interface of the .html calculator coded by the custom GPT. HDR: high-dose rate; LDR: low-dose rate; PSA: prostate specific antigen; PV: prostate volume; G3+3: Gleason 3+3; G3+4: Gleason 3+4; G4+3: Gleason 4+3; G4+4: Gleason 4+4; T1c: stage T1c; T2: stage T2 disease; T2a: stage T2a; T2b: stage T2b; T3: stage T3; T3a: stage T3a; T3b: stage T3b

## Appendices

Appendix A

Appendix B
